# Continuous Monitoring in Implantable Cardiac Devices: Electro-Quasistatic Human Body Communication – Challenges and Opportunities

**DOI:** 10.1109/OJEMB.2026.3722470

**Published:** 2026-08-11

**Authors:** Ayan Biswas, Anyu Jiang, Arunashish Datta, Asif Iftekhar Omi, Baibhab Chatterjee, Shreyas Sen

**Affiliations:** Elmore Family School of Electrical and Computer EngineeringPurdue University 311308 West Lafayette IN 47907 USA; Department of Electrical and Computer EngineeringUniversity of Florida 3463 Gainesville FL 32611 USA; Ixana (Quasistatics Inc.) West Lafayette IN 47906 USA

**Keywords:** Body channel communication, cardiac implantable electronic devices (CIEDs), human body communication, pacemaker longevity, remote patient monitoring, wireless body area networks

## Abstract

Cardiovascular diseases are the leading cause of global mortality, with millions of patients relying on implantable pacemakers. Finite battery energy fundamentally limits remote monitoring. Using open-source manufacturer longevity data, under stated parameter assumptions, we develop an energy-balance model to show a stringent *≈ 130* J/year communication budget, too low for continuous monitoring with conventional RF links. We investigate electro-quasistatic human body communication (EQS-HBC) as an alternative physical layer for pacemakers, combining a systematic review of implant-grade transceivers with a proof-of-concept channel feasibility study in a human-torso phantom. Our analyses indicate that, under these modeling assumptions EQS-HBC could, in principle, support 1 kSps, 16-bit equivalent continuous streaming at $\approx 2\,\mu \mathrm{W}$ ($>\!\!100\times$ more energy-efficient than RF) while remaining within safety standards and preserving $>\!\!14$-year lifetime, outlining design guidance for future implantable cardiac devices.

## I. Introduction

Cardiovascular diseases (CVDs), including ischemic heart disease and stroke, remain the leading cause of mortality worldwide, accounting for over 30% of all deaths [1], [2], [3], [4], [5], [6], [7], [8]. In 2020, more than 500 million individuals were affected globally [9], with nearly 3 million of them requiring implanted pacemakers to manage arrhythmias and heart block conditions. Although modern “smart” pacemakers are central to managing cardiac conditions, their remote connectivity is typically limited to intermittent or event-triggered transmissions because of finite battery capacity and the use of conventional, energy-demanding wireless links [10] to bedside receivers or smartphone applications [11], [12]. These constraints limit timely detection of clinically actionable events and remote patient management [13], [14], [15], [16], [17], [18].

Remote monitoring of cardiac implantable electronic devices (CIEDs) has evolved over five decades (Fig. 1). Transtelephonic monitoring in the 1970 s enabled weekly device checks via telephone landlines [19]. First-generation automatic wireless systems emerged in the 2000 s with Guidant’s Zoom programmer and Medtronic’s CareLink Network, reducing in-office visits by 50-70% [20]. Cloud-integrated platforms followed in the 2010 s, with Biotronik’s Home Monitoring achieving 92% diagnostic accuracy and the REMOTE-CRT study demonstrating non-inferiority to in-office follow-up [20], [21]. Current platforms target integrated AI analytics [22], yet fundamental constraints persist: 14.8-hour average alert latencies [23], 1-1.5 a battery penalties even with sparse transmissions [24], and power budgets that preclude continuous streaming.

Electro-Quasistatic Human Body Communication (EQS-HBC) [25], [26] offers a promising solution to this challenge. EQS-HBC operates in a sub-30 MHz regime where the wavelength in tissue (tens of meters) is much larger than the human body, so signal transmission is governed by quasi-static electric fields rather than radiating waves. In this regime, the body forms a near-field, tissue-coupled channel that can support secure transmission up to 30 Mbps [27], [28], [29] with over 100× lower energy per bit than Bluetooth Low Energy (BLE) [30]. Supplementary Note 1 elaborates more on the fundamentals of EQS-HBC principles governing signal propagation through tissue.

The remainder of this paper is structured as follows: Section II describes the systematic review protocol, energy-balance model, and experimental setup. Section III presents quantitative results from the energy-balance model, experimental channel measurements, and transceiver review. Section IV introduces an EQS-HBC-enabled pacemaker architecture achieving power-parity between sensing and communication, and evaluates its safety profile. Section V concludes as a feasibility assessment study for future deployment of real-time analytics and closed-loop therapy.

## II. Methods

### A. Literature Screening and Benchmarking

A structured literature screening was conducted to identify implant-relevant body-channel and ultra-low-power wireless transceivers for benchmarking EQS-HBC against conventional telemetry. IEEE Xplore and PubMed were searched (2010–2025) using (’body channel communication’ OR ‘human body communication’ OR ’implant transceiver’) AND (’power consumption’ OR ’energy per bit’). Records were screened by title/abstract and then full text using predefined inclusion criteria: (i) integrated Complementary Metal-Oxide-Semiconductor (CMOS) or comparable IC implementation, (ii) reported silicon power and data rate, and (iii) operation below 100 MHz or explicit implant relevance. Fourteen transceivers met these criteria and were used to establish realistic operating points for the energy comparisons and conceptual architecture. Full search details, screening criteria, and extracted parameters are provided in Supplementary Note 2.

### B. Energy-Balance Model

An energy-balance model was developed using Boston Scientific Device Longevity Calculator data [24] for the ESSENTIO™ DR EL pacemaker to estimate the telemetry energy budget compatible with target implant lifetime. Total device energy was separated into baseline implant demand (pacing, sensing, and processing) and communication overhead. Communication feasibility for BLE and EQS-HBC was then estimated using representative energy-per-bit values from the screened literature. These calculations are based on simplifying assumptions regarding pacing workload, battery utilization, and communication duty cycles, and should therefore be interpreted as approximate modeling estimates rather than direct manufacturer-specified telemetry budgets. The governing equations and parameter assumptions are summarized in Supplementary Note 2.

### C. Phantom Channel Characterization

A human-torso phantom was constructed using a Polyethylene Terephthalate (PET) mannequin filled with phosphate-buffered saline (PBS, pH = 7.4) to approximate torso dielectric behavior in the EQS band. A Polydimethylsiloxane (PDMS)-encapsulated implant module was evaluated at 15 anatomically motivated torso locations, while a wrist-mounted receiver emulated an on-body wearable hub. A ring-oscillator-based differential transmitter excited the channel at 20 MHz, and received spectra were recorded for differential and single-ended receiver configurations. Channel gain was computed in dB and summarized across positions to estimate representative and worst-case link margins. Phantom rationale, implant-position definitions, instrumentation settings, and analysis details are given in Supplementary Note 2.

## III. Results

### A. Quantifying the Clinical Monitoring Gap

Analysis of large-scale cardiac device networks reveals critical limitations. A Medtronic CareLink study [23] shows alerts reach clinicians with an average 14.8-hour delay, with 90.9% within 24 hours (Fig. 2(a)). Latency varies by device: Implantable Pulse Generator/Cardiac Resynchronization Therapy Pacemaker (IPG/CRT-P) (17.0–17.2 h), Implantable Cardioverter-Defibrillator (ICD) (13.7 h), Cardiac Resynchronization Therapy Defibrillator (CRT-D) (13.5 h). These delays—averaging one alert daily—highlight power and connectivity constraints precluding continuous monitoring.

**Fig. 1.**
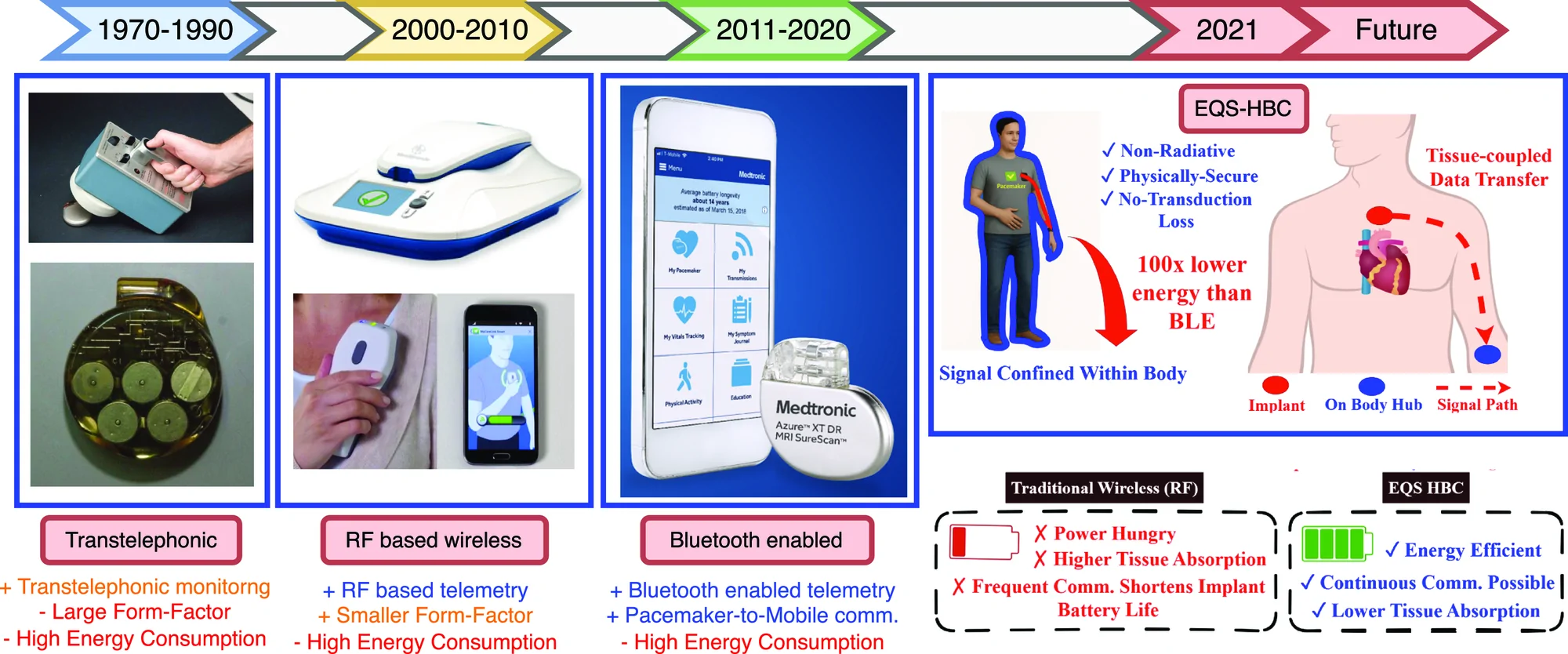
Evolution of Remote Monitoring Techniques for Cardiac Implants.

**Fig. 2.**
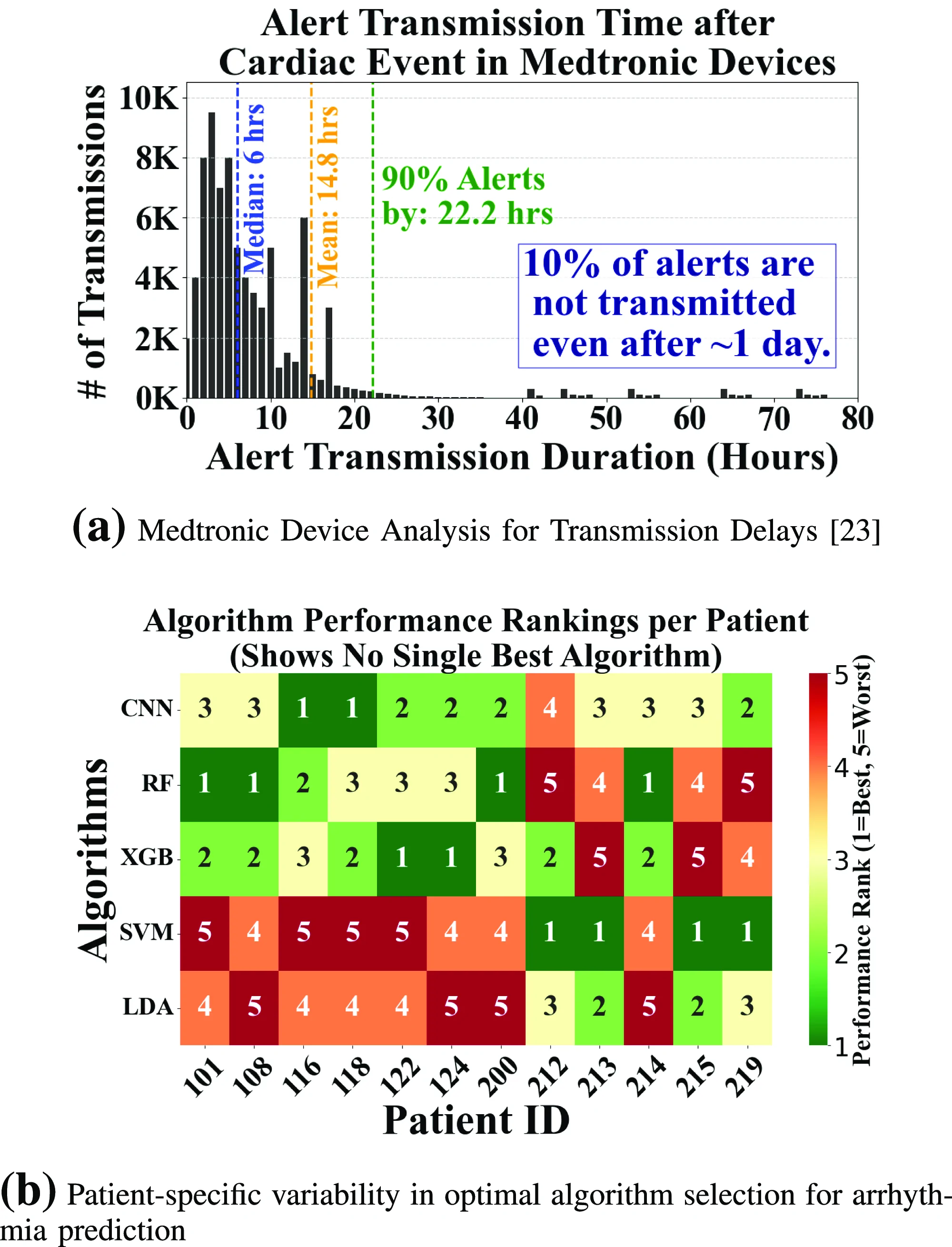
Need for Continuous Remote Monitoring: (a) Medtronic device alert transmission analysis and (b) Patient-specific algorithm variability for arrhythmia detection. (a) Medtronic Device Analysis for Transmission Delays [23] (b) Patient-specific variability in optimal algorithm selection for arrhythmia prediction

Local in-device analytics faces significant challenges. MIT-BIH analysis [31], [32] reveals patient-to-patient heterogeneity in optimal ML algorithms (Fig. 2(a)), requiring patient-specific models—prohibitive for battery-constrained implants. EQS-HBC enables secure raw data transmission to an on-body hub; signals are physically confined to the body [25], ensuring privacy. The hub performs feature extraction and executes individualized ML algorithms, avoiding cloud transmission of raw data. This hybrid approach can deliver personalized arrhythmia detection without complex implant-side logic.

### B. Remote Monitoring in Pacemakers: Current Landscape

Fig. 3(a) shows remote monitoring reduces battery life across Boston Scientific pacemaker families [24] by 1–1.5 years, even with only 1–2 daily transmissions—highlighting communication energy overhead as critical for continuous monitoring.

**Fig. 3.**
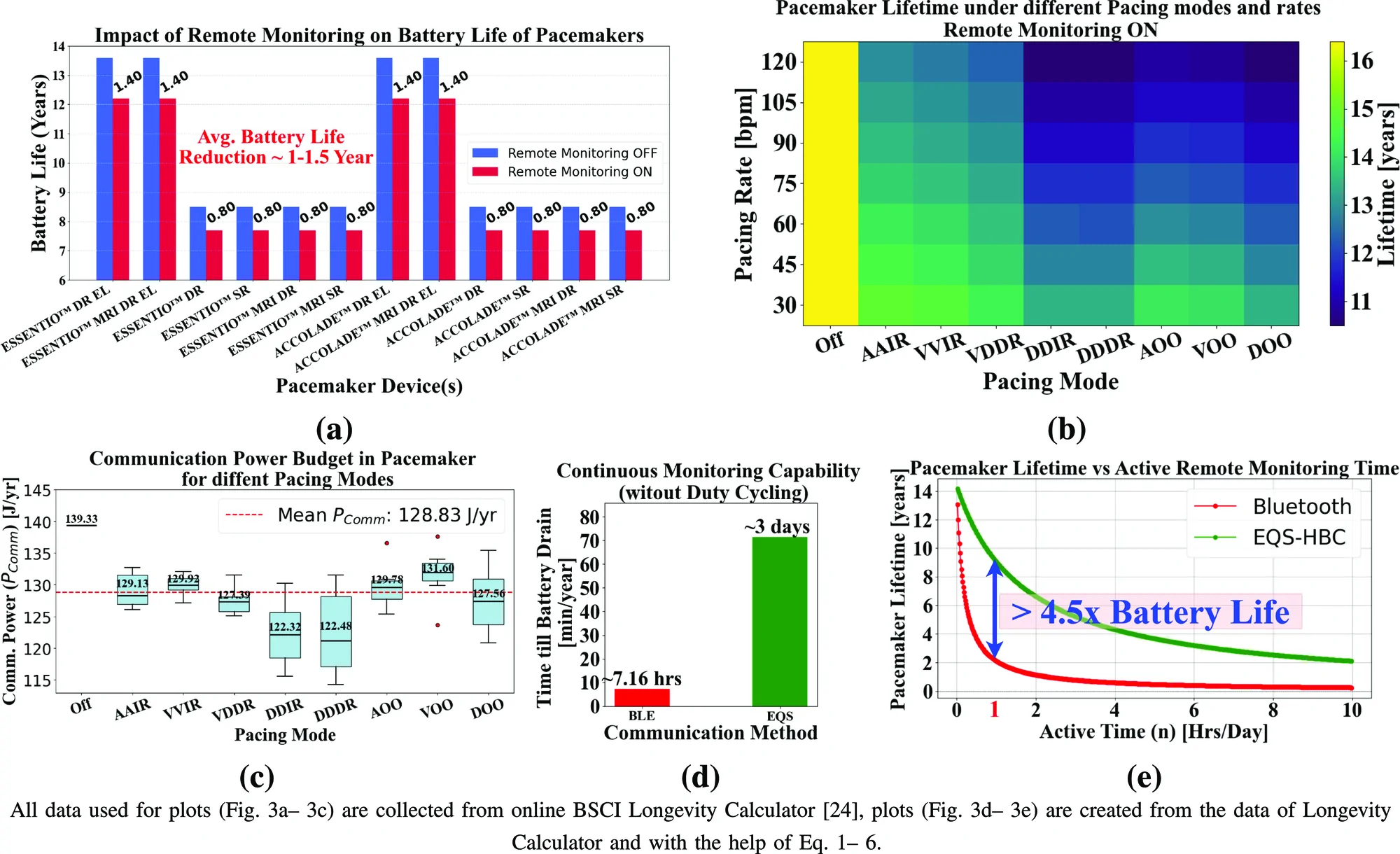
(a) Implant lifetime reduction from remote monitoring (b) ESSENTIO™DR EL lifetime across pacing rates and modes (c) $P_{\rm Comm}$ budget by pacing mode (d) Continuous Monitoring Capability (no duty-cycling) (e) Implant longevity (duty-cycled).

On the other hand, Fig. 3(b) shows complex pacing (DDDR) reduces lifetime from *>*16 years to 11 years, while simpler modes preserve battery life. These trends reinforce the need for highly efficient schemes like EQS-HBC to support continuous monitoring without disproportionate longevity reduction.

### C. Implant Longevity: The Fundamental Challenge

This analysis employs empirical power consumption data for the ESSENTIO™ DR EL pacemaker, obtained from the Boston Scientific Device Longevity Calculator [24], and characterizes how communication overhead impacts projected device lifetime.

The intrinsic power demand of the pacemaker—driven by pacing, sensing, and onboard signal processing—establishes a baseline energy floor. Under typical pacing and sensing configurations, the implant consumes an average energy of $E_{\mathrm{Implant}} = 33.5\,\mu \mathrm{J}$ per heartbeat (as per [24]). Extrapolating this per-cycle consumption across a full year of continuous operation yields the annual implant power budget:

\begin{equation*}
 \begin{split} P_{\mathrm{Implant}} &= E_{\mathrm{Implant}} \times \text{(seconds per year)} \\
 &= 33.5 \,\mu \mathrm{J} \times (365 \times 24 \times 3600) \,\mathrm{s} \\
 &= 1057 \,\mathrm{J/year}. \end{split} \tag{1} 
\end{equation*}

The available energy capacity is determined by the battery chemistry and form factor. For the ESSENTIO™ DR EL platform, a 1.6 A-hr lithium cell operating at a nominal voltage of 2.8 V provides a total energy reserve of

\begin{equation*}
 \begin{split} \mathrm{BatteryCapacity} &= V_{\mathrm{nom}} \times Q_{\mathrm{capacity}} \times t_{\mathrm{conversion}} \\
 &= \mathrm{2.8}\,\mathrm{V} \times 1.6\,\mathrm{A-hr} \times 3600\,\mathrm{s/hr} \\
 &= 16185 \,\mathrm{J}. \end{split} \tag{2} 
\end{equation*}

Given the fixed battery energy and fixed intrinsic implant power demand, device lifetime is inversely proportional to the total power consumption—the sum of baseline implant demand and communication overhead:

\begin{equation*}
 \begin{split} \mathrm{Lifetime}_{\mathrm{Implant}} &= \frac{\mathrm{BatteryCapacity}}{P_{\mathrm{Implant}} + P_{\mathrm{Comm}}}, \end{split} \tag{3} 
\end{equation*}

where $P_{\mathrm{Comm}}$ denotes the power required to sustain remote monitoring at a specified transmission frequency or duty cycle. This relationship reveals a critical insight: any increase in $P_{\mathrm{Comm}}$ produces a multiplicative reduction in device lifetime. Rearranging Eq. (3) isolates the communication power budget—the maximum power that can be allocated to telemetry while maintaining a target device longevity:

\begin{equation*}
 \begin{split} P_{\mathrm{Comm}} &= \frac{\mathrm{BatteryCapacity}}{\mathrm{Lifetime}_{\mathrm{Implant}}} - P_{\mathrm{Implant}}. \end{split} \tag{4} 
\end{equation*}

Using manufacturer-reported lifetimes for each pacing mode and heart rate setting, we computed $P_{\mathrm{Comm}}$ across the range of clinically relevant configurations via Eq. (4). The results, presented in Fig. 3, reveal substantial heterogeneity in the available communication budget depending on pacing mode and rate. Across all modes, the mean available communication power is 128.8 J/year (shown in Fig. 3(c)). Critically, this figure represents the total energy that can be devoted to wireless telemetry while preserving the clinical target longevity of approximately 10–15 years.

Conventional wireless modalities such as BLE, which employ modulation and demodulation in the Industrial, Scientific, and Medical (ISM) band, incur substantial energy overhead for radio frequency synthesis, transduction loss across tissue, and signal processing. When the available communication budget is divided by the energy-per-bit efficiency of current RF systems (typically 10–100 pJ/bit), the result is a communication capacity measured in tens of megabytes per year—insufficient for even modest continuous or near-continuous monitoring scenarios. Current RF-based pacemakers are restricted to infrequent transmissions (1-2 times per day), yielding event-driven rather than continuous surveillance. This intermittent monitoring fundamentally limits the clinical utility of remote monitoring.

The energy budget constraint, therefore, represents the central impediment to transforming pacemaker remote monitoring from episodic to truly continuous. To enable real-time surveillance without sacrificing device longevity, the communication modality itself must achieve orders-of-magnitude improvements in energy efficiency relative to conventional RF approaches. This necessity motivates the exploration of alternative physical layers, such as EQS-HBC, that leverage the human body’s favorable propagation characteristics to achieve energy efficiency while maintaining the functionality and longevity that patients and clinicians depend upon.

### D. Projected Lifetime Gains With EQS-HBC

Conventional RF-based telemetry fundamentally limits how aggressively remote monitoring can be employed in pacemakers because the associated communication power directly competes with the energy required for life-sustaining pacing. Even when transmissions are scheduled infrequently, RF links must overcome tissue attenuation and support carrier generation and RF front-end operation, leading to milliwatt-level average communication power and forcing manufacturers to restrict monitoring to sparse, event-driven uplinks rather than genuinely continuous data streaming [24]. As a result, any substantial increase in transmission frequency rapidly degrades implant longevity, making it impractical to achieve real-time monitoring without compromising the primary pacing function.

To quantify the potential benefit of alternative physical layers, we consider a communication power budget of 128.8 J/year, derived from the previously established analysis of Boston Scientific pacemakers [24]. Within this fixed budget, the maximum continuous communication time supported by a given modality with average power consumption $E_{\alpha }$ is

\begin{equation*}
 \mathrm{Lifetime}_{\alpha } = \frac{P_{\mathrm{Comm}}}{E_{\alpha }}, \quad \alpha \in \lbrace \mathrm{BLE},\, \mathrm{EQS\mathrm{-}HBC}\rbrace, \tag{5} 
\end{equation*}

where $P_{\mathrm{Comm}}$ denotes the available communication power. Using representative values of $E_{\mathrm{BLE}} = \mathrm{5}\,\mathrm{mW}$ for BLE and $E_{\mathrm{EQS\mathrm{-}HBC}} = 500\,\mu \mathrm{W}$ for EQS-HBC, Fig. 3(d) shows that EQS-HBC can sustain continuous telemetry for more than an order of magnitude longer duration than BLE under the same energy constraint. This improvement arises from operating in the EQS regime, which eliminates high-frequency RF carrier generation and leverages the human body as a low-loss channel, thereby reducing both transduction and radiation losses.

In practice, remote monitoring is typically duty-cycled rather than strictly continuous. If remote communication is active for *n* hours per day, the resulting implant lifetime can be expressed as

\begin{equation*}
 \begin{split} \mathrm{Lifetime}_{\mathrm{Duty-Cycled}} = \frac{\mathrm{BatteryCapacity}}{P_{\mathrm{Implant}} + n P_{\mathrm{Comm}}}, \end{split} \tag{6} 
\end{equation*}

where $P_{\mathrm{Implant}}$ is the baseline pacing and sensing power. As illustrated in Fig. 3(e), under the assumed parameters, EQS-HBC yields approximately a $3\mathrm{--}5\times$ higher effective device lifetime compared to BLE-class telemetry for the same monitoring intensity. These estimates depend on assumptions regarding energy-per-bit, duty cycle, and packet size, and should therefore be interpreted as indicative margins rather than precise predictions. Within these modeling constraints, EQS-HBC suggests a promising physical-layer pathway toward enabling more frequent or near-continuous monitoring with reduced impact on device longevity. However, realizing this potential in practice will require comprehensive system-level optimization and experimental validation.

### E. Channel Measurements: EQS-HBC Link Characterisation

We experimentally validated the EQS-HBC channel using a human-torso phantom with a PDMS-encapsulated prototype implant (6 cm × 3.5 cm × 2 cm) [33] submerged in PBS (pH = 7.4) within a PET mannequin (Fig. 4 a). A reconfigurable ring oscillator (made using SN74AHC04BQAR inverters) generated differential excitation (1–30 MHz); an external spectrum analyzer measured channel loss at the wrist for differential and single-ended configurations.

**Fig. 4.**
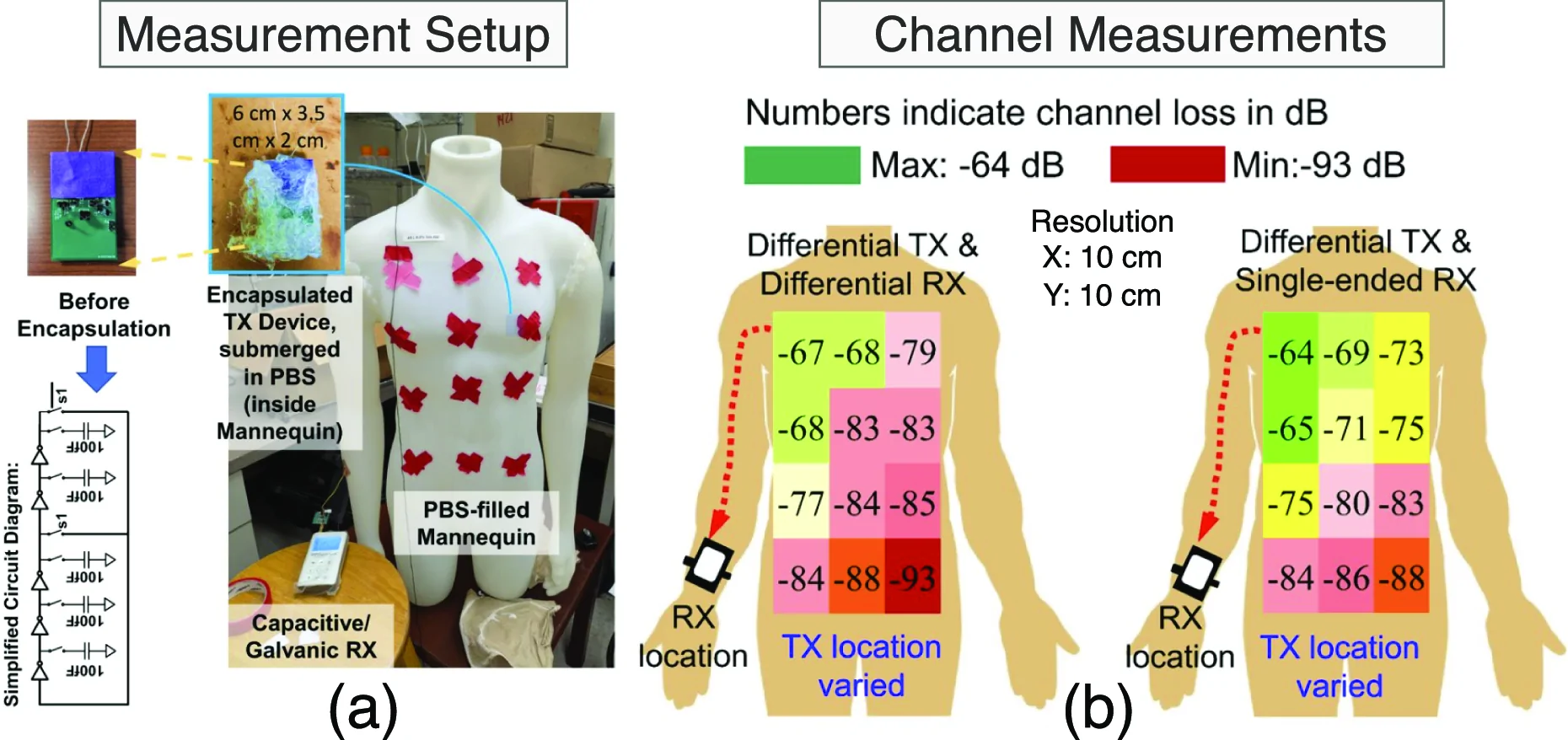
Measured results of the Channel Gain (CG) at Electro-Quasistatic (EQS) region (at 20 MHz) in a mannequin are shown. —(a) The setup includes a Polydimethylsiloxane (PDMS)-encapsulated differential transmitter (TX) implant submerged in phosphate-buffered saline (PBS) inside the mannequin. The TX, implemented as a ring oscillator (tuned at 20 MHz), is illustrated on the left. (b) The CG (in dB) observed by both a differential receiver (RX) and a single-ended RX placed at the wrist is presented for various TX locations.

Channel loss (shown in Fig. 4(b)) varied from −67 dB (right shoulder) to −93 dB (left waist), with the heart-implant-to-wrist path exhibiting −83 dB loss—well within receiver sensitivity. Single-ended reception outperformed differential by 2–4 dB, favoring wearable hub integration. The flat response across 1–30 MHz confirms wideband communication (1 kSps, 16-bit) is feasible without equalisation. These measurements validate link budget assumptions, confirming EQS-HBC as a stable propagation medium for ultra-low-power transceivers.

### F. Implant-Grade Transceivers: A Review

Prior works [32], [43] demonstrate a clear progression in body channel communication (BCC) transceiver design from standards-compliant systems to ultra-low-power implant applications. Table I summarizes this evolution, spanning early IEEE 802.15.6-compliant designs achieving 90 pJ/bit efficiency [34] to subsequent high-data-rate implementations reaching 11–140 pJ/bit at 5–80 Mbps [35], [36], [37], [38]. Efficiency breakthroughs through magnetic HBC and optimized OOK further reduced energy to 7.15–41 pJ/bit [39], [40].

**TABLE I:** State-of-The-Art Implant-Grade and Body Channel Communication Transceivers

**Reference**	**Technology**	**Data Rate**	**Power**	**Energy/bit**	**Application**
*Standards-Compliant HBC*					
Jang 2019 [34]	180 nm CMOS	10 Mbps	900 *μ*W	90 pJ/bit	IEEE 802.15.6
*High-Data-Rate HBC*					
Bae 2011 [35]	130 nm CMOS	5 Mbps	700 *μ*W (Rx)	140 pJ/bit (Tx)	Body Sensor Networks
Mercier 2011 [36]	65 nm CMOS	10 Mbps	110 *μ*W	11 pJ/bit	eTextiles
Saadeh 2017 [37]	65 nm CMOS	2 Mbps	1.1 mW	550 pJ/bit	OFDM
Maity 2019 [29]	65 nm CMOS	30 Mbps	–	6.3 pJ/bit	Interference-Robust HBC
Jang 2019 [38]	65 nm CMOS	80 Mbps	*< 1* mW (Tx)	12.5 pJ/bit	Capsule Endoscopy
*Ultra-Low-Power EQS-HBC*					
Park 2019 [39]	65 nm CMOS	5 Mbps	35.8 *μ*W	7.15 pJ/bit	Magnetic HBC
Zhao 2019 [40]	40 nm CMOS	4 Mbps	164 *μ*W	41 pJ/bit	OOK
**Maity 2020** [41]	**65 nm CMOS**	**10 kbps**	**415 nW**	**41.5 pJ/bit**	**Secure EQS**
**Chatterjee 2023** [42]	**65 nm CMOS**	**1 Mbps**	**0.52 *μ*W**	**0.52 pJ/bit**	**Brain Implant**
*Commercial Reference*					
BLE (Typical)	-	1 Mbps	5-10 mW	5-10 nJ/bit	Wearables

Note: Energy/bit calculated at maximum data rate. EQS-HBC implementations shown in bold.

This evolution culminates in EQS-HBC implementations achieving sub-*μ*W total power consumption—the first physically secure node consuming only 415 nW at 10 kbps [41] and biphasic quasistatic brain implants reaching 0.52 *μ*W at 1 Mbps [42]. These represent ${>}100\times$ improvement over conventional BLE (5–10 nJ/bit), confirming EQS-HBC as the enabling technology for continuous implant monitoring. Recent passive galvanic HBC further eliminates transmit overhead [44], setting the stage for next-generation implant longevity.

## IV. Discussion: EQS-HBC-Enabled Architecture for Continuous Monitoring

Building on the established energy constraints of conventional RF telemetry, this section details how EQS-HBC provides a viable pathway to continuous monitoring. We present a conceptual system architecture for an EQS-HBC-enabled pacemaker and evaluate its safety profile, demonstrating how this paradigm overcomes the fundamental limitations of current approaches.

### A. EQS-HBC Enabled Pacemaker: Conceptual System Design

Fig. 5(a) illustrates a conceptual architecture for continuous, millisecond-resolution physiological monitoring — enabled by tissue-coupled electro-quasistatic human body communication (EQS-HBC). The system can achieve 1 KSps sampling (16-bit resolution) with a sensing duration $T_{\mathrm{sense}} \approx 18$ s, generating approximately 286 Kbit of raw physiological data that—accounting for protocol overhead—fits within a modest 48 KB on-chip memory buffer. This buffered data is then transmitted in a compact burst using a 5 Mbps EQS-HBC link consuming only 500 *μ*W active power (equivalent to 100 pJ/bit energy efficiency), completing the communication phase $T_{\mathrm{comm}} \approx 80$ ms.

**Fig. 5.**
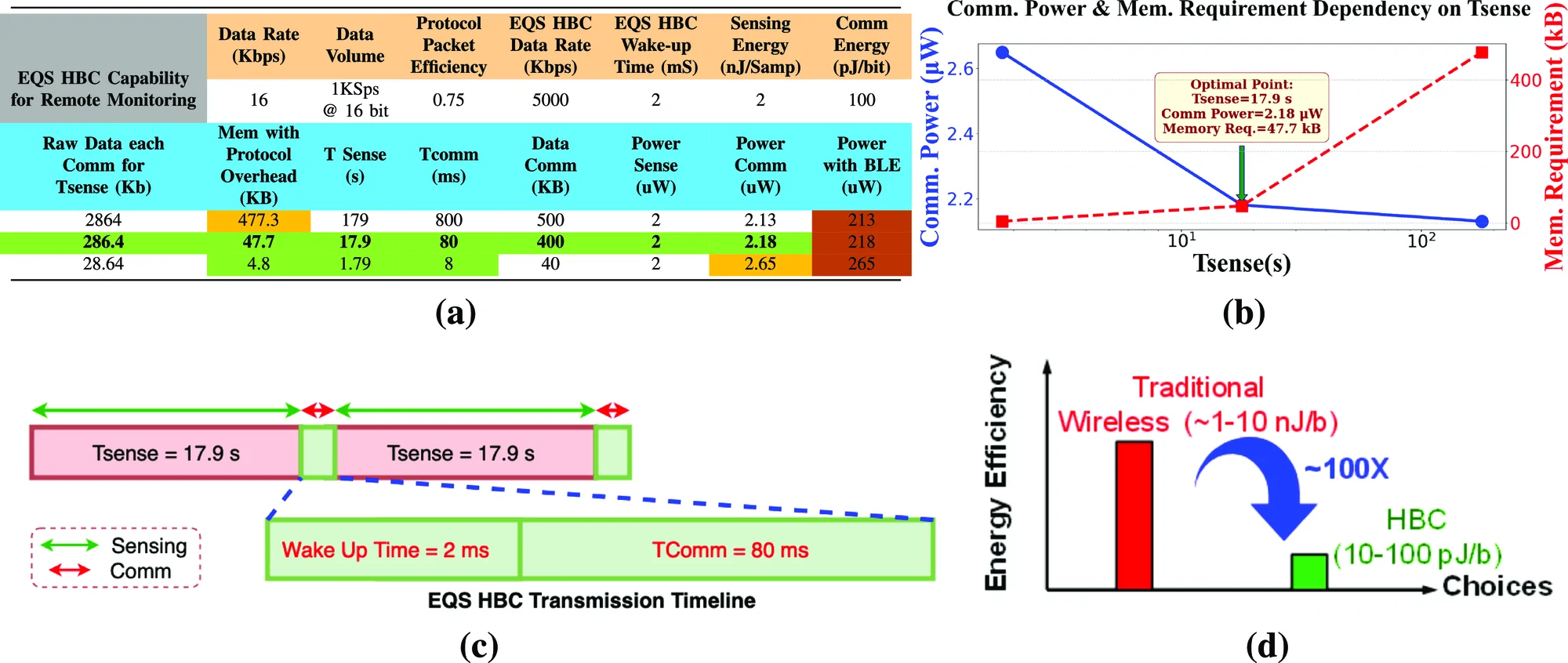
(a) Table: EQS-HBC features and memory/power analysis (b) Optimal sensing/communication point (c) Sensing and communication timeline (d) Energy efficiency: Traditional wireless vs. HBC

Including transceiver wake-up overhead of 2 ms, the resulting average communication power for continuous 1 KSps monitoring is just 2.18 *μ*W—directly comparable to the sensing power budget of approximately 2 *μ*W achievable with state-of-the-art analog-to-digital converters operating at 2 nJ/sample (or 30 fJ/conversion-step). This power parity represents a fundamental breakthrough: continuous high-fidelity data acquisition becomes energetically feasible without compromising the primary pacing functionality of the implant.

Such millisecond-granularity continuous monitoring would be categorically impossible using conventional Bluetooth Low Energy (BLE) telemetry. Equivalent BLE transmission of the same data volume would demand an average communication power of approximately 218 *μ*W—more than two orders of magnitude higher—resulting in unacceptably rapid battery depletion that would render the device clinically inviable within months rather than the required 10–15 a service life.

The system design optimizes the interplay between sensing duration $T_{\mathrm{sense}}$ and communication burst efficiency. Reducing $T_{\mathrm{sense}}$ by 10× would minimize on-chip memory requirements and associated leakage power, but would extend $T_{\mathrm{comm}}$ to approach the transceiver wake-up time (2 ms), thereby degrading average communication efficiency. Conversely, extending $T_{\mathrm{sense}}$ by 10× would necessitate substantially larger memory arrays with prohibitive leakage currents. The selected $T_{\mathrm{sense}} = 17.9$ s represents the optimal design point, delivering continuous fine-grain (every millisecond) physiological data with only 18 s end-to-end latency using just 48 KB on-chip memory, while maintaining perfectly balanced sensing and communication power budgets of approximately 2 *μ*W each. This elegant architecture demonstrates how EQS-HBC fundamentally resolves the longstanding tension between continuous monitoring utility and implantable device longevity constraints.

### B. Safety Assessment and Regulatory Implications

Safe operation within established electromagnetic exposure limits is critical for any implant-to-wearable telemetry scheme. For the galvanic EQS-HBC link considered here, we directly evaluated in-body electric fields and tissue heating under a representative implant excitation of $V_{\mathrm{TX}} = 0.15$ V$_{pp}$ using a tissue-equivalent phantom and finite-element modeling (Fig. 6). Fig. 6(a) shows the measured in-body electric-field distribution, which decays radially away from the transmitter. The maximum in-tissue field is $5.8 \times 10^{-2}\,\mathrm{V/m}$, approximately *1069×* lower than the International Commission on Non-Ionizing Radiation Protection (ICNIRP) in-tissue limit of 62 $\mathrm{V/m}$ in the relevant frequency range.

**Fig. 6.**
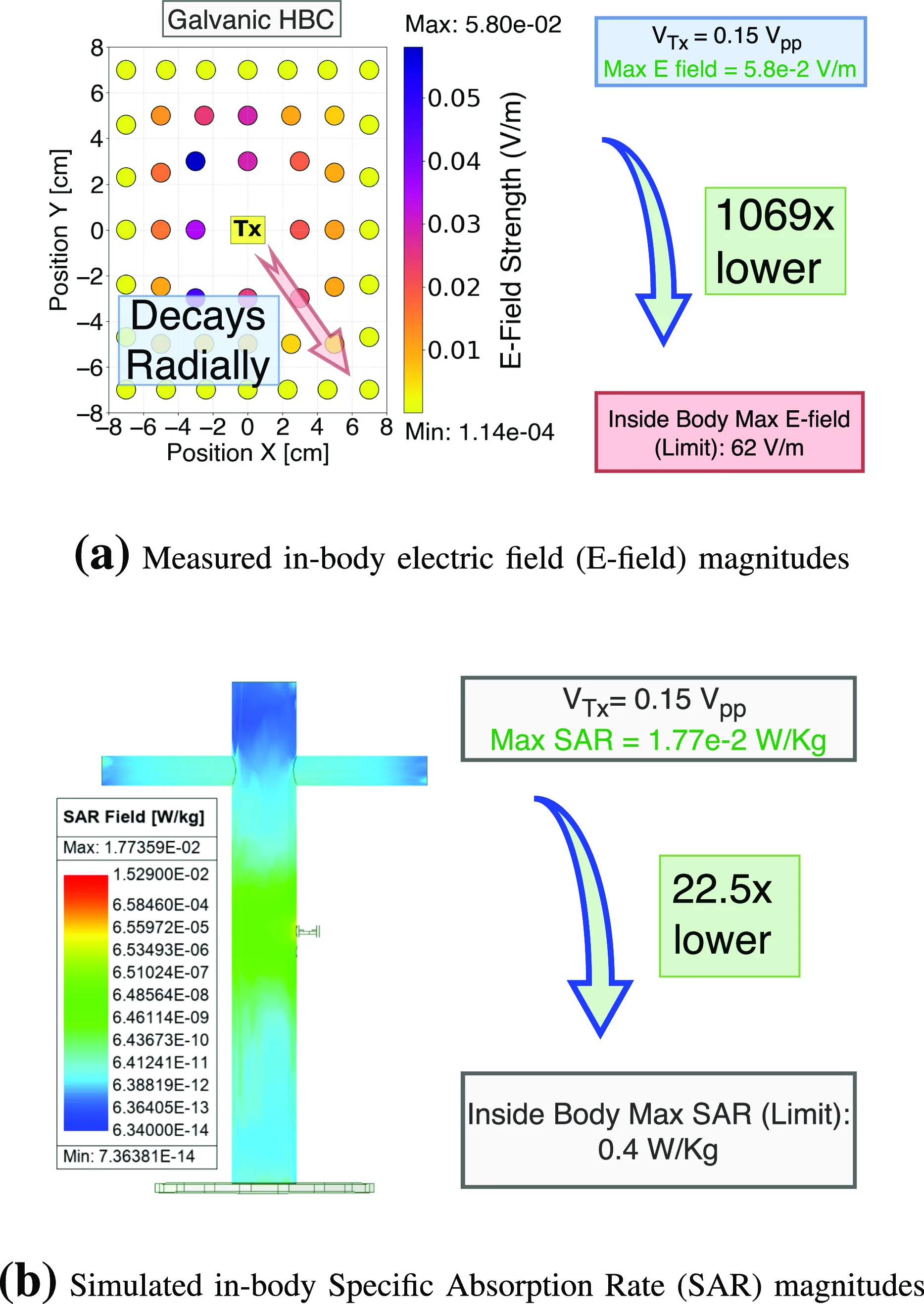
Safety assessment of EQS-HBC: (a) Measured in-body electric-field (E-field) magnitudes and (b) Simulated 10 g-averaged SAR distribution for a representative galvanic EQS-HBC implant excitation ($V_{\mathrm{TX}} = 0.15$ V$_{pp}$). Both remain well below ICNIRP exposure limits [45], [46], [47] across the operating frequency range. (a) Measured in-body electric field (E-field) magnitudes (b) Simulated in-body Specific Absorption Rate (SAR) magnitudes

Fig. 6(b) reports the corresponding 10 g-averaged Specific Absorption Rate (SAR) distribution obtained from the same excitation. The peak SAR is $1.77\times 10^{-2}\,\mathrm{W/kg}$, which is about *22.5×* below the ICNIRP local limit of 0.4 $\mathrm{W/kg}$. These measurements confirm that, for the drive levels required by our conceptual EQS-HBC architecture, both electric-field exposure and tissue heating remain well within internationally accepted ICNIRP [27], [45], [46], [47] safety guidelines. These results are consistent with prior biophysical analyses [26], [48], [49], [50] of EQS-HBC, where coupling is predominantly electro-quasistatic and fields are confined near the electrodes, leading to low bulk tissue absorption. Detailed descriptions of the phantom model, field-probe calibration, SAR extraction procedure, and comparisons to previously reported HBC safety studies are provided in Supplementary Note 3 and are intended to complement the system-level safety analysis presented here.

## V. Conclusion and Future Direction

In this study, we theoretically analyzed how EQS-HBC can act as a promising physical layer to address the energy, safety, and channel challenges that currently prevent true continuous monitoring in cardiac implants. Current RF telemetry—with milliwatt power, 14.8-hour alert latencies [23], and 1–1.5 a battery penalties—necessitates event-driven operation, limiting proactive care. Our modeling suggests that EQS-HBC could, in principle, achieve ${>}100\times$ energy efficiency (100 pJ/bit), operate ${>}100\times$ below ICNIRP limits [27], [45], [46], [47], and use body-coupled propagation to support 1 KSps continuous monitoring with 2 *μ* W balanced sensing/communication budgets and 48KB memory (Fig. 5). Lifetime analysis further indicates 4.5–10× longer projected longevity for 1 h/day monitoring (*>*14 years vs. 2–3 years for BLE) (Figs. 3(d)–3(e)) [24], leveraging mature transceivers (415 nW nodes, 30 Mbps links [29], [30], [42]). However, translating this potential into practice will require future work on the implementation of a complete EQS-HBC–enabled cardiac system and comprehensive pre-clinical validation before any deployment in patients.

## Supplementary Materials

The Supplementary Materials provide EQS-HBC theory, detailed methods, and safety analysis and exposure results in this work.

Supplementary Materials

## References

[1] Institute for Health Metrics and Evaluation, “Global burden of disease 2021: Findings from the GBD 2021 study,” Seattle, WA, Tech. Rep. 2024. [Online]. Available:https://www.healthdata.org/research-analysis/library/global-burden-disease-2021-findings-gbd-2021-study

[2] National Center for Health Statistics, “Multiple cause of death 2018–2022 on CDC wonder database,” 2022. [Online]. Available: https://wonder.cdc.gov/mcd.html

[3] American Heart Association, “2024 heart disease and stroke statistics: A report of us and global data from the american heart association,” 2024, doi: 10.1161/CIR.000000000000120.

[4] J. A. Wolfram and J. K. Donahue, “Gene therapy to treat cardiovascular disease,” J. Amer. Heart Assoc., vol. 2, no. 4, 2013, Art. no. e000119. 10.1161/JAHA.113.000119 PMC3828796 23963752

[5] G. A. Mensah and D. W. Brown, “An overview of cardiovascular disease burden in the United States,” Health Affairs, vol. 26, no. 1, pp. 38–48, 2007. 10.1377/hlthaff.26.1.38 17211012

[6] World Health Organization, “Cardiovascular diseases,” 2025. [Online]. Available: https://www.who.int/news-room/fact-sheets/detail/cardiovascular-diseases-(cvds)

[7] P. A. Heidenreich et al., “Forecasting the future of cardiovascular disease in the United States: A policy statement from the american heart association,” Circulation, vol. 123, no. 8, pp. 933–944, 2011. 10.1161/CIR.0b013e31820a55f5 21262990

[8] A. Kumar, D. DiPette, and J. D. Potts, “Novel approaches for treating cardiac disease,” Microsc. Microanalysis, vol. 25, no. S2, pp. 1188–1189, 2019.

[9] Centers for Disease Control and Prevention, “Global responses to prevent, manage, and control CVDS,” 2022. Accessed: Jun. 2, 2025. [Online]. Available: https://www.cdc.gov/pcd/issues/2022/22_0347.htm

[10] A. Zurbuchen et al., “The swiss approach for a heartbeat-driven lead- and batteryless pacemaker,” Heart Rhythm, vol. 14, no. 2, pp. 294–299, Feb. 2017, doi: 10.1016/j.hrthm.2016.10.016. 27756706

[11] K. G. Tarakji et al., “Performance of first pacemaker to use smart device app for remote monitoring,” Heart Rhythm O2, vol. 2, no. 5, pp. 463–471, Oct. 2021, doi: 10.1016/j.hroo.2021.07.008. PMC8505204 34667961

[12] Medtronic, “Pacing systems,” 2025. Accessed: Jun. 2, 2025. [Online]. Available: https://www.medtronic.com/se-sv/healthcare-professionals/products/cardiac-rhythm/pacemakers.html

[13] B. Vandenberk and S. R. Raj, “Remote patient monitoring: What have we learned and where are we going?,” Curr. Cardiovasc. Risk Rep., vol. 17, no. 6, pp. 103–115, Apr. 2023, doi: 10.1016/j.hroo.2021.07.008. PMC10122094 37305214

[14] G. Y. Lip et al., “European heart rhythm association/heart failure association joint consensus document on arrhythmias in heart failure, endorsed by the heart rhythm society and the asia pacific heart rhythm society,” Europace, vol. 18, no. 1, pp. 12–36, Aug. 2015, doi: 10.1093/europace/euv191. 26297713

[15] H. Burri and D. Senouf, “Remote monitoring and follow-up of pacemakers and implantable cardioverter defibrillators,” Europace, vol. 11, no. 6, pp. 701–709, May 2009, doi: 10.1093/europace/eup110. PMC2686319 19470595

[16] B. Ruyobeza, S. S. Grobbelaar, and A. Botha, “Hurdles to developing and scaling remote patients’ health management tools and systems: A scoping review,” Systematic Rev., vol. 11, no. 1, Aug. 2022, doi: 10.1186/s13643-022-02033-z. PMC9427160 36042505

[17] H. Heidbuchel, “Telemonitoring of implantable cardiac devices: Hurdles towards personalised medicine,” Heart, vol. 97, no. 11, pp. 931–939, May 2011, doi: 10.1136/hrt.2009.188789. 21558069

[18] R. Abdolkhani, K. Gray, A. Borda, and R. DeSouza, “Patient-generated health data management and quality challenges in remote patient monitoring,” JAMIA Open, vol. 2, no. 4, pp. 471–478, Sep. 2019, doi: 10.1093/jamiaopen/ooz036. PMC6993998 32025644

[19] N. M. Hemel, “Remote monitoring of implanted cardiac devices: A plea for a nationwide exploration,” Neth. Heart J., vol. 17, no. 11, pp. 434–437, 2009. [Online]. Available: https://pmc.ncbi.nlm.nih.gov/articles/PMC2779481/10.1007/BF03086298 PMC2779481 19949713

[20] N. Varma and C. Love, “Remote monitoring of cardiac implantable electronic devices: What is the evidence?,” J. Arrhythmia, 2023. [Online]. Available: https://pmc.ncbi.nlm.nih.gov/articles/PMC9877501/

[21] ScienceSoft, “Remote cardiac monitoring: Benefits, use cases, costs,” 2024. [Online]. Available: https://www.scnsoft.com/healthcare/medical-devices/remote-cardiac-monitoring

[22] I. Antoun et al., “Artificial intelligence and the future of cardiac implantable electronic devices: Diagnostics, monitoring, and therapy,” J. Clin. Med., vol. 14, no. 24, Dec. 2025. Art. no. 8824, doi: 10.3390/jcm14248824. PMC12733696 41464726

[23] E. M. Cronin, J. C. Green, J. Lande, T. R. Holmes, D. Lexcen, and T. Taigen, “Performance of alert transmissions from cardiac implantable electronic devices to the carelink network: A retrospective analysis,” Cardiovasc. Digit. Health J., vol. 4, no. 3, pp. 72–79, Jun. 2023. doi: 10.1016/j.cvdhj.2023.03.003. PMC10282010 37351335

[24] B. Scientific, “Device longevity calculator,” 2025, Accessed: Jun. 30, 2025. [Online].Available: https://www.bostonscientific.com/en-EU/medical-specialties/electrophysiology/device-longevity/longevity-calculator.html

[25] D. Das, S. Maity, B. Chatterjee, and S. Sen, “Enabling covert body area network using electro-quasistatic human body communication,” Sci. Rep., vol. 9, no. 1, Mar. 2019, Art. no. 4160, doi: 10.1038/s41598-018-38303-x. PMC6411898 30858385

[26] N. Modak, M. Nath, B. Chatterjee, S. Maity, and S. Sen, “Bio-physical modeling of galvanic human body communication in electro-quasistatic regime,” IEEE Trans. Biomed. Eng., vol. 69, no. 12, pp. 3717–3727, Dec. 2022. 10.1109/TBME.2022.3176541 35594211

[27] S. Maity, M. Nath, G. Bhattacharya, B. Chatterjee, and S. Sen, “On the safety of human body communication,” IEEE Trans. Biomed. Eng., vol. 67, no. 12, pp. 3392–3402, Dec. 2020. 10.1109/TBME.2020.2986464 32305887

[28] S. Maity, B. Chatterjee, G. Chang, and S. Sen, “A 6.3pj/b 30mbps -30db sir-tolerant broadband interference-robust human body communication transceiver using time domain signal-interference separation,” in Proc. Custom Integr. Circuits Conf., 2018, pp. 1–4.

[29] S. Maity, B. Chatterjee, G. Chang, and S. Sen, “Bodywire: A 6.3-pj/b 30-mb/s -30-db sir-tolerant broadband interference-robust human body communication transceiver using time domain interference rejection,” IEEE J. Solid-State Circuits, vol. 54, no. 10, pp. 2892–2906, Oct. 2019.

[30] S. Maity et al., “A 415 nw physically and mathematically secure electro-quasistatic HBC node in 65 nm CMOS for authentication and medical applications,” in Proc. Custom Integr. Circuits Conf., 2020, pp. 1–4.

[31] G. Moody and R. Mark, “Mit-bih arrhythmia database,” 2025, Accessed: Jun. 2, 2025. [Online].Available: https://www.physionet.org/content/mitdb/1.0.0/

[32] A. Biswas, B. Chatterjee, and S. Sen, “Enabling high temporal-resolution remote monitoring in resource-constrained implantable medical devices with human body communication,” in Proc. 21st Int. Conf. Body Sensor Netw., Nov. 2025, pp. 1–4, doi: 10.1109/BSN66969.2025.11337643.

[33] A. Jiang, D. Suarez, A. Biswas, B. Chatterjee, and S. Sen, “Channel measurements for ultra-low-power tissue-coupled communication in personalized cardiac pacing and continuous monitoring,” in Proc. Microw., Antennas, Propag. Conf., 2025, pp. 1–4.

[34] J. Jang, H. Cho, and H.-J. Yoo, “An 802.15.6 HBC standard compatible transceiver and 90 pj/b full-duplex transceiver for body channel communication,” in Proc. Biomed. Circuits Syst. Conf., 2019, pp. 1–4.

[35] J. Bae, L. Yan, and H.-J. Yoo, “A low energy injection-locked FSK transceiver with frequency-to-amplitude conversion for body sensor applications,” IEEE J. Solid-State Circuits, vol. 46, no. 4, pp. 928–937, Apr. 2011.

[36] P. P. Mercier and A. P. Chandrakasan, “A supply-rail-coupled etextiles transceiver for body-area networks,” IEEE J. Solid-State Circuits, vol. 46, no. 6, pp. 1284–1295, Jun. 2011.

[37] W. Saadeh, M. A. B. Altaf, H. Alsuradi, and J. Yoo, “A 1.1-mW ground effect-resilient body-coupled communication transceiver with pseudo OFDM for head and body area network,” IEEE J. Solid-State Circuits, vol. 52, no. 10, pp. 2690–2702, Oct. 2017.

[38] J. Jang et al., “A four-camera VGA-resolution capsule endoscope system with 80-mb/s body channel communication transceiver and sub-centimeter range capsule localization,” IEEE J. Solid-State Circuits, vol. 54, no. 2, pp. 538–549, Feb. 2019.

[39] J. Park and P. P. Mercier, “A sub-10-pj/bit 5-mb/s magnetic human body communication transceiver,” IEEE J. Solid-State Circuits, vol. 54, no. 11, pp. 3031–3042, Nov. 2019.

[40] J. Zhao et al., “A 4-Mbps 41-pj/bit on-off keying transceiver for body-channel communication with enhanced auto loss compensation technique,” in Proc. Asian Solid-State Circuits Conf., 2019, pp. 173–176.

[41] S. Maity et al., “Sub-*μ*wrcomm: 415-nw 1–10-kb/s physically and mathematically secure electro-quasi-static HBC node for authentication and medical applications,” IEEE J. Solid-State Circuits, vol. 56, no. 3, pp. 788–802, Mar. 2021.

[42] B. Chatterjee et al., “Biphasic quasistatic brain communication for energy-efficient wireless neural implants,” Nature Electron., vol. 6, no. 9, pp. 703–716, Aug. 2023, doi: 10.1038/s41928-023-01000-3.

[43] Q. Huang et al., “Human body communication transceivers,” Nature Rev. Elect. Eng., vol. 2, no. 6, pp. 374–389, Mar. 2025, doi: 10.1038/s44287-025-00160-y.

[44] Y. Chang, W. Wang, Y. Zou, and K. Yang, “RPG-HBC: Reconfigurable passive galvanic human body communication for bioelectronic implants under varying channel conditions,” in Proc. Custom Integr. Circuits Conf., 2025, pp. 1–3.

[45] International commission on non-ionizing radiation protection, “ICNIRP guidelines for limiting exposure to time-varying electric, magnetic and electromagnetic fields (up to 300 GHz),” 1998, Accessed: Feb. 8, 2024. [Online].Available: https://www.icnirp.org/cms/upload/publications/ICNIRPemfgdl.pdf 9525427

[46] IEEE Standard for Safety Levels with Respect to Human Exposure to Radio Frequency Electromagnetic Fields, 3 kHz to 300 GHz, IEEE Standard C95.1-2005 (Revision of IEEE Standard C95.1-1991), Inst. Elect. Electron. Engineers, New York, NY, USA, Apr. 2006.

[47] IEEE Standard for Safety Levels with Respect to Human Exposure to Radio Frequency Electromagnetic Fields, 3 kHz to 300 GHz, IEEE Standard IEEE C95.1-1991, New York, NY, USA, 2002. [Online].Available: https://www.sandiegocounty.gov/content/dam/sdc/pds/ceqa/Soitec-Documents/Final-EIR-Files/references/rtcref/ch9.0/RTCrefappx/2014-12-19_IEEE2002.pdf

[48] A. Datta, M. Nath, D. Yang, and S. Sen, “Advanced biophysical model to capture channel variability for EQS capacitive HBC,” IEEE Trans. Biomed. Eng., vol. 68, no. 11, pp. 3435–3446, Nov. 2021. 10.1109/TBME.2021.3074138 33872142

[49] A. Biswas, S. Sarkar, B. Chatterjee, and S. Sen, “Is in-body to out-of-body communication primarily inter-device coupling?,” in Proc. Microw., Antennas, Propag. Conf., 2025, pp. 1–4.

[50] A. Biswas, “Electro quasistatic human body communication for implant to wearable links: Biophysical modeling and system level exploration,” Master’s thesis, Purdue University, West Lafayette, IN, USA, 2026, doi: 10.25394/PGS.33087698. [Online]. Available: https://hammer.purdue.edu/articles/thesis/ELECTRO_QUASISTATIC_HUMAN_BODY_COMMUNICATION_FOR_IMPLANT_TO_WEARABLE_LINKS_BIOPHYSICAL_MODELING_AND_SYSTEM_LEVEL_EXPLORATION/33087698

