# Supplementary material for: Continuous Monitoring in Implantable Cardiac Devices: Electro-Quasistatic Human Body Communication – Challenges and Opportunities
Source: IEEE Open J Eng Med Biol. 2026 Aug 11;7:324–31. doi: 10.1109/OJEMB.2026.3722470 (PMC13667299; doi:10.1109/OJEMB.2026.3722470)
Supplement: Supplementary file 1 [file supp1-3722470.pdf]

Continuous Monitoring in Implantable Cardiac Devices: Electro-Quasistatic Human Body Communication – Challenges and Opportunities

Ayan Biswas, *Graduate Student Member, IEEE,* Anyu Jiang, *Graduate Student Member, IEEE,* Arunashish Datta, *Member, IEEE,* Asif Iftekhar Omi, *Graduate Student Member, IEEE,* Baibhab Chatterjee, *Member, IEEE,* and Shreyas Sen, *Senior Member, IEEE*

I. Supplementary Note 1

Fundamentals of Electro-Quasistatic Human Body Communication

Electro-Quasistatic Human Body Communication (EQS-HBC) operates in a frequency regime where the electromagnetic wavelength in tissue is much larger than the relevant body and implant dimensions. In this regime, the fields around the body can be treated as quasi-static: they vary in time but do not form fully developed propagating waves over the length of the channel [1, 2]. This approximation provides physical insight [3] into why EQS-HBC can enable low-power, body-coupled communication for implantable devices [4, 5].

The total electric field $\vec{E}$ can be decomposed into a dominant electro-quasistatic component $\vec{E}_{\mathrm{EQS}}$ and a small error term $\vec{E}_{\mathrm{error}}$ that accounts for higher-order wave effects:

$$\vec{E} = \vec{E}_{\mathrm{EQS}} + \vec{E}_{\mathrm{error}}.$$

A standard estimate for the relative error in the electro-quasistatic approximation is given by

$$\frac{|\vec{E}_{\mathrm{error}}|}{|\vec{E}|} \approx \omega^2 \mu_{\mathrm{tissue}} \varepsilon_{\mathrm{tissue}} r^2,$$

where $\omega = 2\pi f$ is the angular frequency, μ_{tissue} and $\varepsilon_{\mathrm{tissue}}$ are the effective permeability and permittivity of tissue, and r is a characteristic length scale (e.g., the size of the electrodes or local body dimension near the implant) [2, 6, 7]. For the electro-quasistatic approximation to hold, this dimensionless quantity must be much less than one.

For millimeter-scale electrodes with $r < 1$ cm and tissue permittivity on the order of $10^3 \varepsilon_{\mathrm{air}}$ across the MHz range [8, 9], the condition

$$\omega^2 \mu_{\mathrm{tissue}} \varepsilon_{\mathrm{tissue}} r^2 \ll 1$$

translates into a frequency constraint of approximately

$$f \ll \frac{1}{2\pi r \sqrt{\mu_{\mathrm{tissue}} \varepsilon_{\mathrm{tissue}}}} \approx 150 \text{ MHz},$$

for representative tissue parameters and implant dimensions [2]. Operating well below this upper bound keeps the electro-quasistatic error small and ensures that $\vec{E} \approx \vec{E}_{\mathrm{EQS}}$ throughout the domain of interest.

In this work, all analysis and measurements are therefore restricted to:

$$f \leq 20 \text{ MHz},$$

which maintains the relative error at only a few percent (<4%) for typical tissue properties and length scales [2]. Within this sub-20 MHz regime, the fields around the body are dominated by near-field, quasi-static behavior, and radiated components make a negligible contribution to the implant-to-wearable link.

II. Supplementary Note 2

Methods: Literature Screening, Energy-Balance Model, Phantom Design

A. Literature Screening and Benchmarking Details

The literature screening was designed to assemble a compact but representative set of integrated transceivers for benchmarking EQS-HBC against conventional low-power wireless links. Searches in IEEE Xplore and PubMed (2010–2025) using `('body channel communication' OR 'human body communication' OR 'implant transceiver') AND ('power consumption' OR 'energy per bit')` returned 87 unique records after duplicate removal. Only English-language journal and conference papers were considered to ensure access to full technical descriptions and measurement data.

Title and abstract screening removed papers that clearly did not involve integrated circuits, low-power communication, or body-related channels. The remaining articles underwent full-text review, where the inclusion criteria were: (i) implementation in integrated CMOS or a comparable IC process, (ii) explicit reporting of measured silicon power consumption and data rate, and (iii) operation below 100 MHz or explicit implant or implant-to-wearable relevance. Simulation-only works, system-level demonstrations without transceiver power figures, and purely wearable-to-wearable links without any implant context were excluded. Fourteen transceivers satisfied all criteria. For each, we extracted technology node, operating frequency, signaling or modulation method, maximum data rate, total power consumption, and reported or derived energy-per-bit, which are tabulated in Table I in the main manuscript. These devices provide realistic operating points for the energy comparisons in Fig. 3 and support the conceptual architecture

in Fig. 4 of main manuscript by demonstrating that sub-pJ/bit to few-pJ/bit body-channel links have been achieved in silicon.

B. Energy-Balance Model Parameters and Sensitivity

The energy-balance model in Section III (in main manuscript) uses the ESSENTIO™ DR EL pacemaker as a representative platform to estimate how much energy can be devoted to telemetry while preserving typical longevity. Manufacturer data from the Boston Scientific Device Longevity Calculator [10] provide nominal battery capacity, assumed pacing conditions, and projected lifetime. Baseline annual implant energy is obtained by multiplying the manufacturer-reported per-beat energy by the number of heartbeats per year at the nominal pacing rate. Battery energy is computed from the nominal voltage and rated capacity. The difference between the total battery energy and baseline implant consumption over the target lifetime yields the communication budget that can be allocated to telemetry without violating longevity constraints.

For comparing BLE and EQS-HBC, the annual communication energy is modeled as the product of the number of transmitted bits and the energy-per-bit, including protocol overhead [11]. Representative BLE energy-per-bit values (5–10 nJ/bit) and EQS-HBC values (sub-pJ/bit to a few pJ/bit) are taken from the transceivers identified in Table I of the main manuscript. Here we note that sensitivity analysis was performed by varying energy-per-bit, the number of monitoring hours per day, and the amount of data per monitoring interval over realistic ranges. Across these variations, the qualitative conclusions remain unchanged: under the same longevity constraints, EQS-HBC accommodates substantially more frequent or continuous telemetry than BLE-class RF links before exhausting the available communication budget.

C. Phantom Design, Implant Positions, and Channel Analysis

The phantom study in Section IV was intended to test whether an EQS-HBC link between a pacemaker-like implant and a wrist-worn hub remains feasible across a range of realistic implant locations. A commercial PET mannequin was selected as a rigid torso shell, and filled with phosphate-buffered saline (PBS, pH = 7.4) to approximate bulk torso conductivity and permittivity in the 20 MHz band. The PBS mixture provides a homogeneous medium that simplifies interpretation of channel variations with position while capturing first-order loading effects. The implant module was encapsulated in PDMS prior to immersion, providing electrical isolation from the saline and serving as a simplified analog of the insulating outer boundary of a packaged cardiac device.

Fifteen implant positions were defined to span clinically motivated pockets and deeper locations, including left and right pre-pectoral regions, upper and lower anterior torso sites, lateral placements, and more central positions approximating heart-adjacent implants. Each position was assigned a distinct label and marked on the mannequin to allow repeatable placement, while the wrist receiver location was kept fixed. At each position, a ring-oscillator-based differential transmitter [12] excited the channel at 20 MHz, and the received spectrum was measured for both differential and single-ended receiver configurations. Channel gain was computed in dB as the ratio of received to transmitted voltage. To summarize spatial variability, we report representative and worst-case gains across the 15 positions rather than performing formal hypothesis testing, reflecting the goal of establishing feasibility bounds and worst-case link margins. The observed spread in gain is compared against reported receiver sensitivities from prior body-channel transceivers, confirms that, even under conservative placement, the measured EQS-HBC channel remains compatible with practical low-power receivers.

III. SUPPLEMENTARY NOTE 3

SAFETY ASSESSMENT : MODELING BASIS, MEASUREMENT SETUP, AND RELATION TO PRIOR WORK

The main text summarizes safety at the level appropriate for the manuscript body by reporting the peak in-tissue electric field and 10 g-averaged specific absorption rate (SAR) for the representative EQS-HBC implant excitation used in this study. This supplementary note provides the additional technical basis for that summary, including the modeling framework, measurement setup, and the relationship between the present safety analysis and prior EQS-HBC safety studies. The intent is not to claim a complete device-level regulatory evaluation, but to establish that the proposed implant-to-wrist EQS-HBC operating conditions remain well within accepted electromagnetic exposure limits and are consistent with previously reported electro-quasistatic safety trends.

At electro-quasistatic frequencies, safety is governed primarily by local electric-field exposure and the corresponding SAR, rather than by far-field radiation metrics. Prior biophysical studies of HBC have shown that, in the EQS regime, the dominant fields are localized around the electrodes and decay rapidly with distance, while magnetic coupling and radiative loss remain negligible [1, 2, 13]. These characteristics are favorable for implant telemetry because tissue heating is limited by the small excitation amplitudes and the confinement of the quasi-static field. In addition, previous implant-to-wearable EQS-HBC modeling has shown that channel transfer is dominated by tissue permittivity rather than conductivity, so safety-relevant field strength depends more strongly on electrode geometry and spatial placement than on RF-style propagation effects [2, 14].

A. FEM simulation framework :

To evaluate safety under implant-relevant operating conditions, finite-element electromagnetic simulations were performed in ANSYS HFSS using a simplified cross-cylindrical human model [13] previously validated against anatomically detailed voxel models [15] for EQS-HBC analysis. The model consists of a 1.8 m tall cylindrical tissue volume with an effective muscle radius of 13.6 cm and a superficial skin layer, which together provide a computationally efficient but physiologically grounded approximation for low-frequency field analysis. For the SAR study, the implanted transmitter was placed 5 cm below the skin surface and driven at 150 mV_{pp}, corresponding closely to the representative transmitter excitation used in the present work. A rubber isolation layer was

included beneath the body model to represent body-to-ground capacitance and avoid unrealistically strong ground coupling in the simulation environment.

The SAR distribution was computed using the IEEE/IEC 62704-1 standard [16]. Under galvanic EQS-HBC excitation, the simulated peak 10-g-averaged SAR was 1.77×10^{-2} W/kg. Compared with the ICNIRP 2020 local SAR basic restriction [17] for general-public exposure in the head and torso (2 W/kg), this value is approximately 113× lower. In addition to SAR, the spatial electric-field distribution was extracted to quantify in-tissue exposure directly. The maximum electric field under the same excitation was 5.8×10^{-2} V/m, which is approximately 1069× below the in-tissue ICNIRP electric-field limit of 62 V/m. These values form the basis of Figure 6 in the main manuscript.

B. Experimental measurement setup :

To complement the simulation analysis, in-tissue field measurements were performed in a porcine-tissue phantom using the same low-voltage excitation scale. Fresh pork tissue was selected because its dielectric properties in the 5–30 MHz range approximate those of human muscle, making it a practical surrogate for localized EQS field measurements. The tissue properties were independently characterized using a two-port vector network analyzer and 3.5 mm coaxial probes inserted to 2 cm depth. At 17 MHz, the extracted relative permittivity and conductivity were $\varepsilon_r = 68.2 \pm 2.1$ and $\sigma = 0.88 \pm 0.05$ S/m, respectively, in close agreement with established muscle reference data.

The EQS-HBC measurement platform employed an implant-sized galvanic transmitter with differential excitation and an on-body receiver representative of the implant-to-wearable use case. For field measurements, a handheld spectrum analyzer together with calibrated near-field probes was used to quantify local electric- and magnetic-field magnitudes inside the tissue phantom under a transmitter excitation of 0.15 V_{pp}. The measured electric-field map exhibited radial decay away from the transmitter, with a peak value of 5.8×10^{-2} V/m, matching the value reported in the main-text figure. The measured field can be converted to SAR using the standard tissue relation $SAR = \sigma |E|^2 / \rho$, yielding values consistent with the simulation-based safety margin. All tissue samples were obtained from a commercially licensed food supplier; no live animals were used, and no IACUC approval was required.

C. Interpretation and scope :

Figure 6 in main manuscript therefore combines two complementary pieces of evidence tied to the proposed EQS-HBC operating regime: measured in-tissue electric-field exposure in tissue phantom and simulated SAR under matched low-voltage implant excitation. This is still appropriately limited in scope. Specifically, the present analysis does not yet constitute a full regulatory safety dossier for a finished pacemaker implementation, since it does not include implant-package-specific materialization, chronic encapsulation effects, patient-specific anatomical heterogeneity, lead interactions, or long-duration in-vivo thermal studies.

These limitations are consistent with the scope of the paper as a preclinical feasibility study. Accordingly, the conclusions drawn from the safety analysis are intentionally limited to the following claim: for the representative galvanic EQS-HBC implant excitation considered here, both local electric-field exposure and SAR remain comfortably below ICNIRP [18] and IEEE [19, 20] safety limits, and no immediate barrier is evident from first-order electromagnetic safety metrics.

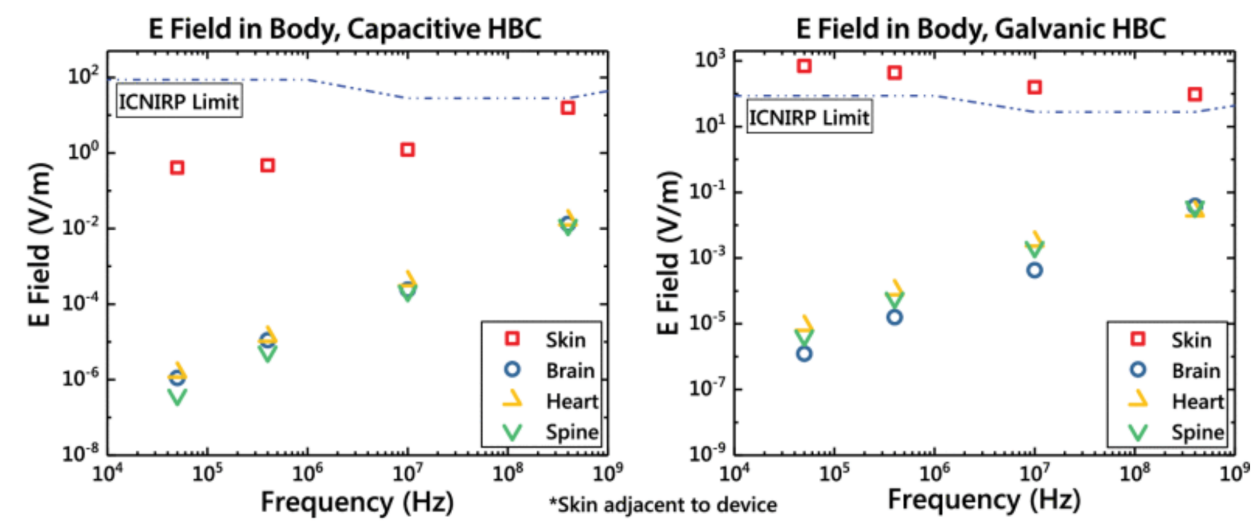

(a) Simulated in-body electric field (E-field) magnitudes

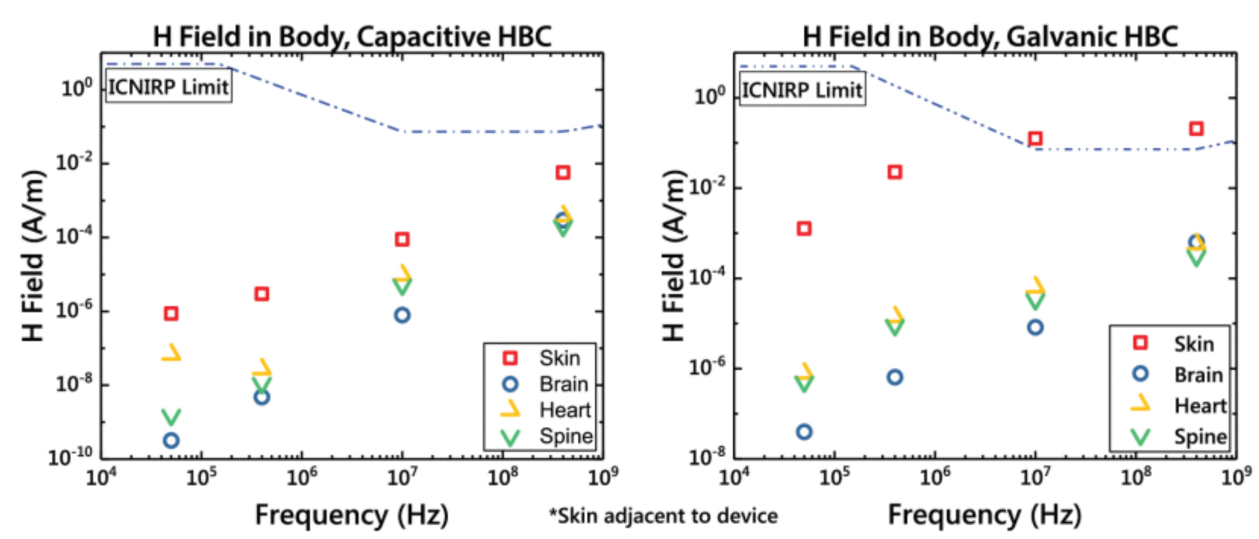

(b) Simulated in-body magnetic field (H-field) magnitudes

Fig. 1. Safety assessment of EQS-HBC [13]: simulated E-field (a) and H-field (b) distributions in different tissues for capacitive and galvanic HBC remain well below ICNIRP exposure limits [18–20] over the operating frequency range, confirming safe operation for implantable and wearable use.

Prior EQS-HBC safety analyses [1, 13] (as summarized in Fig. 1) and the present phantom and simulation results are therefore mutually supportive: the earlier works establish the general electro-quasistatic safety rationale, while the measurements and simulations reported here explicitly tie that rationale to the specific implant-to-wrist communication scenario considered in this manuscript.

REFERENCES

[1] D. Das et al., "Enabling covert body area network using electro-quasistatic human body communication", *Scientific Reports*, vol. 9, no. 1, Mar. 2019.
[2] N. Modak et al., "Bio-physical modeling of galvanic human body communication in electro-quasistatic regime", *IEEE Transactions on Biomedical Engineering*, vol. 69, no. 12, 2022.
[3] A. Biswas et al., "Is in-body to out-of-body communication primarily inter-device coupling?", in *2025 IEEE Microwaves, Antennas, and Propagation Conference (MAPCON)*, 2025.
[4] B. Chatterjee et al., "Biphasic quasistatic brain communication for energy-efficient wireless neural implants", *Nature Electronics*, vol. 6, no. 9, Aug. 2023.
[5] A. Biswas, "Electro quasistatic human body communication for implant to wearable links: Biophysical modeling and system level exploration", M.S. thesis, 2026.
[6] MIT OpenCourseWare, *Lecture notes: Electromagnetic energy: From motors to lasers*, MIT OpenCourseWare, Massachusetts Institute of Technology, Accessed: May 04, 2026.
[7] J. Larsson, "Electromagnetics from a quasistatic perspective", *American Journal of Physics*, vol. 75, no. 3, Mar. 2007.

[8] IT'IS Foundation, *Tissue properties from Gabriel-Gabriel model*, Online database, Available at: https://itis.swiss/virtual-population/tissue-properties/database/dielectric-properties/. Accessed: December 25, 2025, 2021.
[9] S. Gabriel et al., "The dielectric properties of biological tissues: Iii. parametric models for the dielectric spectrum of tissues", *Physics in Medicine and Biology*, vol. 41, no. 11, Nov. 1996.
[10] Boston Scientific, *Device longevity calculator*, https://www.bostonscientific.com/en-EU/medical-specialties/electrophysiology/device-longevity/longevity-calculator.html, Accessed: 2025-06-30.
[11] A. Biswas et al., "Enabling high temporal-resolution remote monitoring in resource-constrained implantable medical devices with human body communication", in *2025 IEEE 21st International Conference on Body Sensor Networks (BSN)*, Nov. 2025.
[12] A. Jiang et al., "Channel measurements for ultra-low-power tissue-coupled communication in personalized cardiac pacing and continuous monitoring", in *2025 IEEE Microwaves, Antennas, and Propagation Conference (MAPCON)*, 2025.
[13] S. Maity et al., "On the safety of human body communication", *IEEE Transactions on Biomedical Engineering*, vol. 67, no. 12, 2020.
[14] A. Datta et al., "Advanced biophysical model to capture channel variability for eqs capacitive hbc", *IEEE Transactions on Biomedical Engineering*, vol. 68, no. 11, 2021.
[15] Neva Electromagnetics, *Vhp female model v2.2*, Accessed Nov 25, 2025, 2025.
[16] *Iec/ieee international standard for determining the peak spatial average specific absorption rate (sar) in the human body from wireless communications devices, 30 mhz to 6 ghz. part 1: General requirements for using the finite difference time domain (fdtd) method for sar calculations*, Published 2017-10-27, International Electrotechnical Commission, Institute of Electrical, and Electronics Engineers, 2017.
[17] International Commission on Non-Ionizing Radiation Protection (ICNIRP), "Guidelines for limiting exposure to electromagnetic fields (100 khz to 300 ghz)", *Health Physics*, vol. 118, no. 5, May 2020.
[18] International Commission on Non-Ionizing Radiation Protection (ICNIRP), *ICNIRP Guidelines for Limiting Exposure to Time-Varying Electric, Magnetic and Electromagnetic Fields (up to 300 GHz)*, https://www.icnirp.org/cms/upload/publications/ICNIRPemfgdl.pdf, [Online; accessed 8-Feb-2024], 1998.
[19] "Ieee standard for safety levels with respect to human exposure to radio frequency electromagnetic fields, 3 khz to 300 ghz", *IEEE Std C95.1-2005 (Revision of IEEE Std C95.1-1991)*, 2006.
[20] *IEEE Standard for Safety Levels With Respect to Human Exposure to Electromagnetic Fields, 0–3 kHz*, https://www.sandiegocounty.gov/content/dam/sdc/pds/ceqa/Soitec-Documents/Final-EIR-Files/references/rtcref/ch9.0/RTCrefappx/2014-12-19_IEEE2002.pdf, [Online; accessed 8-Feb-2024], IEEE, 2002.
